# pCLE highlights distinctive vascular patterns in early gastric cancer and in gastric diseases with high risk of malignant complications

**DOI:** 10.1038/s41598-021-00550-w

**Published:** 2021-10-26

**Authors:** Mara Fornasarig, Alessandra Capuano, Stefania Maiero, Eliana Pivetta, Giovanni Guarnieri, Vincenzo Canzonieri, Antonella Zucchetto, Maurizio Mongiat, Renato Cannizzaro, Paola Spessotto

**Affiliations:** 1 Oncological Gastroenterology - Centro di Riferimento Oncologico di Aviano (CRO), IRCCS, Aviano, Italy; 2 Molecular Oncology - Centro di Riferimento Oncologico di Aviano (CRO), IRCCS, Aviano, Italy; 3 Pathology - Centro di Riferimento Oncologico di Aviano (CRO), IRCCS, Aviano, Italy; 4 Department of Medical, Surgical and Health Sciences - University of Trieste, Trieste, Italy; 5 Unit of Cancer Epidemiology - Centro di Riferimento Oncologico di Aviano (CRO), IRCCS, Aviano, Italy

**Keywords:** Cancer, Gastroenterology, Oncology, Risk factors

## Abstract

Endoscopy is widely used to detect and diagnose precancerous lesions and gastric cancer (GC). The probe-based Confocal Laser Endomicroscopy (pCLE) is an endoscopic technique suitable for subcellular resolution and for microvasculature analyses. The aim of this study was to use pCLE to identify specific vascular patterns in high-risk and early stage GC. Mucosal architecture, vessel tortuosity, enlargements and leakage were assessed in patients with autoimmune gastritis and early gastric cancer (EGC). We were able to stratify gastritis patients by identifying distinct vascular profiles: gastritis was usually associated with increased vascularization characterized by a high number of tortuous vessels, which were also found in atrophic autoimmune disease. Leaky and tortuous vessels, distributed in a spatially irregular network, characterized the atrophic metaplastic mucosa. The mucosal vasculature of EGC patients displayed tortuous vessels, but unlike what detected in atrophic gastritis, they appeared patchy, as is in neoplastic gastric tissue. Very importantly, we detected vascular changes even in areas without lesions, supporting the contention that vascular alterations may provide a favorable microenvironment for carcinogenesis. This report confirms that pCLE is a valid endoscopic approach to improve the definition of patients with malignant lesions or at increased risk for GC by assessing vascular changes.

## Introduction

Gastric cancer (GC) is the fifth leading cause of cancer deaths. Data from 2019 Italian Cancer Registry show a 5-year mortality of 32% for GC, likely due to delayed diagnosis at advanced stages of the disease^1^. Detection of GC at early stages is challenging in Western countries, although the development of GC is a consequence of a long evolution of precancerous lesions. Gastric atrophy is an important step in the path to GC, starting with chronic active gastritis leading to glandular loss and the development of atrophic gastritis, followed by intestinal metaplasia and dysplasia, which eventually develops into the intestinal type of gastric adenocarcinoma, as described by Correa in 1975^2,3^.

Two of the known clinical conditions leading to gastric atrophy are *Helicobacter pylori* infection and autoimmune gastritis caused by organ-specific antibodies such as anti-parietal cell antibodies^4^. In addition, autoimmune gastritis has also been associated with the development of neuroendocrine tumors of the stomach^5,6^. Macroscopic features of gastric atrophy, represented by atrophic and enlarged folds and swellings of a pale-colored mucosa, are easily seen on endoscopy of the upper gastrointestinal tract^7^.

One of the main challenges in improving GC survival is the introduction and development of new and less invasive tools for early detection. The often inconspicuous appearance of pre- and early carcinomas can present a challenge for endoscopic visualization with white light^8^. Advances in endoscopic techniques and the use of dye solutions with final image enhancement have helped the endoscopist to better distinguish normal from dysplastic lesions^9–11^.

Recently, confocal laser endomicroscopy technology, which increases the contrast and magnification of diseased tissue at the cellular level, offers promise for improving endoscopic diagnostic capability^12^. The probe-based Confocal Laser Endomicroscopy (pCLE) enables in vivo analysis of tissue microarchitecture and precise identification of areas suitable for biopsy sampling^12,13^. High-resolution confocal imaging is achieved by i.v. injection of fluorescein and allows the endoscopist to visualize cellular and subcellular structures as well as capillaries and flowing erythrocytes in vivo^14^. Thus, the technology provides both structural and functional information, as leakage of dye from the less efficient vessels can be detected and measured. Indeed, vascular permeability has been assessed using this approach in patients with ulcerative colitis^15^. This technique has also been used in a few studies to objectively assess microvessel density at various neoplastic stages^16–18^ and angiogenic status of gastrointestinal cancers^19–21^. Indeed, pCLE has been shown to be not only a useful diagnostic tool, but also a valid technique for evaluating vascular changes in GC^21,22^.

In this study, we used the pCLE technique to examine the vasculature associated with endoscopically normal mucosa in lesions with malignant phenotype or increased risk of GC, with the aim of verifying whether inflammatory, preneoplastic conditions or early GC (EGC) have particular vascular alterations.

## Results

### Patients’ classification

Patients were divided in five groups as reported in Tables 1 and 2 and based on the following characteristics:

- Four healthy people (“1–4n”) displaying a regular gastric mucosa without any features of gastritis.
- Eleven patients (“1–11g”) with mild gastritis as defined by OLGA-OLGIM^23^.
- Seven patients (“1–7aag”) in surveillance for atrophic autoimmune gastritis.
- Twelve patients (“1–12amg”) in surveillance for atrophic metaplastic autoimmune gastritis. Metaplasia was localized at the corpus-fundus with antral sparing.
- Eight patients with EGC or high-grade dysplasia ("1-8EGC") who were further evaluated with pCLE after HD white light and NBI inspection to look for other possible dysplastic/neoplastic foci before planning endoscopic or surgical treatment. Patient #1EGC showed multiple erosions on the antrum and biopsies revealed the presence of high-grade dysplasia. pCLE showed dysplastic foci consistent with only one eroded area removed by endoscopic submucosal dissection (ESD). Patient #2EGC was found to have an adenocarcinoma focus on incidental biopsies obtained during a gastritis workup. pCLE allowed the diagnosis of multifocal GC and gastrectomy was planned as treatment. Targeted pCLE-guided biopsies from a 10 mm excavated lesion (0-III by Paris classification^24^) on the antrum in patient #3EGC allowed the diagnosis of adenocarcinoma rather than high-grade dysplasia. The lesion was surgically removed. Patient #4EGC showed an intraepithelial adenocarcinoma at the angulus and the lesion was removed by ESD. Patient #5EGC was diagnosed with intraepithelial adenocarcinoma based on biopsies taken from a 10 mm sessile pseudopolyp lesion (0-Is). Patient #6EGC was found to have intraepithelial adenocarcinoma within an inflammatory polyp on the antrum and was removed via ESD. Patients #7 and #8ECG underwent surgery for the presence of neoplastic lesions (0-IIc) that were not amenable to ESD treatment.

**Table 1:** Characteristics of gastritis patients.

	Sample	Age	Gender	OLGA-OLGIM stage		*H. pylori* status
				Antrum	Corpus/fundus	
Normal (n)	#1n	51	F	0	0	Negative
	#2n	57	M	0	0	Negative
	#3n	49	M	0	0	Negative
	#4n	32	F	0	0	Negative
Gastritis (g)	#1g	46	M	0	0	Negative
	#2g	49	F	0	0	Negative
	#3g	62	F	0	0	Positive
	#4g	67	M	I	0	Negative
	#5g	65	M	I	0	Negative
	#6g	68	F	0	0	Negative
	#7g	58	M	I	0	Negative
	#8g	65	F	0	0	Negative
	#9g	52	F	0	0	Negative
	#10g	43	F	0	0	Negative
	#11g	67	M	0	0	Negative
Atrophic autoimmune gastritis (aag)	#1aag	70	M	I	II	Negative
	#2aag	46	F	I	II	Negative
	#3aag	69	F	I	III	Negative
	#4aag	56	F	I	III	Negative
	#5aag	53	F	I	II	Negative
	#6aag	46	F	I	III	Negative
	#7aag	48	M	I	III	Negative
Atrophic metaplastic autoimmune gastritis (amg)	#1amg	58	F	I	III	Negative
	#2amg	62	F	II	III	Negative
	#3amg	39	M	II	III	Negative
	#4amg	40	M	I	IV	Negative
	#5amg	70	M	I	IV	Negative
	#6amg	77	F	I	III	Negative
	#7amg	62	F	II	IV	Negative
	#8amg	64	M	I	IV	Negative
	#9amg	52	M	I	IV	Negative
	#10amg	67	M	I	III	Positive
	#11amg	58	F	I	IV	Negative
	#12amg	62	M	I	IV	Negative

**Table 2:** Characteristics of patients with high grade dysplasia or early gastric cancer (EGC).

Sample	Age	Gender	Histology	*H. pylori* status	Site	TNM	Treatment
#1EGC	55	M	High grade dysplasia	Negative	Antrum	NA	Endoscopic
#2EGC	71	M	Intestinal Adenocarcinoma	Negative	Multifocal-antrum and corpus	T1N0M0	Surgery
#3EGC	67	M	Intestinal adenocarcinoma	Negative	Antrum	T1bN0M0	Surgery
#4EGC	77	F	Intestinal adenocarcinoma	Negative	Angulus	T1aN0M0	Endoscopic
#5EGC	72	M	Intestinal adenocarcinoma	Negative	Antrum	T1sN0M0	Endoscopic
#6EGC	76	F	Intestinal adenocarcinoma	Negative	Antrum	T1aN0M0	Endoscopic
#7EGC	59	M	Diffuse adenocarcinoma	Positive	Corpus	T1bN0M0	Surgery
#8EGC	55	F	Intestinal adenocarcinoma	Positive	Antrum	T1bN0M0	Surgery

*NA* not applicable.

### Gastric pit patterns

Using pCLE, we found that the normal mucosa showed round pits and round openings, the pyloric glands showed continuous short rod-shaped pits with slit-like openings, while the inflammatory gastric mucosa showed regular pits with increased fluorescein signal in the stromal compartment; atrophic gastric mucosa showed reduced pits with dilated openings, and the appearance of goblet cells with dark mucin was a typical feature of gastritis associated with intestinal metaplasia. High grade dysplasia was characterized by distorted pits with irregular epithelial lining, while cancer showed atypical glands without regular pits (Table 3).

**Table 3:** Vascular and gastric pit pattern features in patients analyzed by pCLE.

	Total	Type of vessel alterations (pCLE)	Cases/total	Gastric pit pattern (pCLE)	Cases/total
Normal	4	No alterations	**4/4**	Regular pits	**4/4**
Gastritis	11	No alterations	2/11	Regular pits	**11/11**
		Vessel tortuosity	5/11		
		Leakage	1/11		
		Vessel tortuosity + leakage	3/11		
Atrophic autoimmune gastritis	7	Increased density of tortuous vessels	**7/7**	Reduced number of regular pits	**7/7**
Atrophic metaplastic autoimmune gastritis	12	Increased density of tortuous vessels with changes in spatial distribution + leakage	**12/12**	Reduced number of regular pits and presence of goblet cells	**12/12**
Early gastric cancer	8	Vessel tortuosity (irregular distribution)	3/8	Dysplastic (focal)	1/8
		Vessel tortuosity (irregular distribution) + leakage	3/8	Neoplastic (focal)	6/8
		Vessel tortuosity (irregular distribution) and enlargement + leakage	2/8	Neoplastic (multi focal)	1/8

Bold characters highlight that all cases (100%) show the specific vascular alteration/characteristic and the specific gastric pit pattern.

### Vascular patterns

By the use of pCLE, we carefully described the vessel architecture to verify the presence of peculiar and distinctive vascular patterns in patients with inflammation-associated atrophic, non atrophic autoimmune gastritis, and EGC. Specifically, we looked for the presence of leakage, vessel tortuosity and enlargement, which are considered solid criteria for characterizing vascular alterations, as previously described^19–21^ and reported in the Methods section.

**Figure 1:**
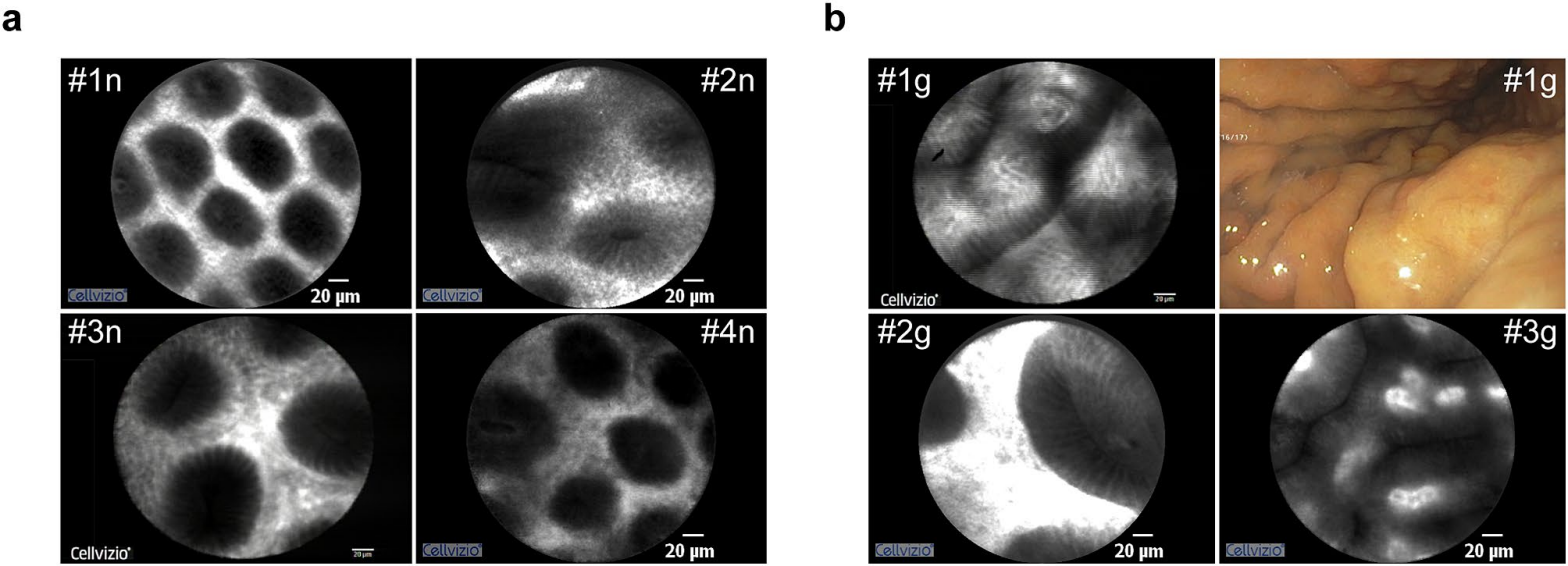
pCLE images of the normal gastric mucosa (“n”) (**a**) and mild gastritis (“g”) (**b**). Images of patients #1–3g are reported as examples of the features of gastric mucosa in gastritis. A representative endoscopic image of non atrophic gastric mucosa is also shown.

The pCLE analyses provide clear images of the microvascular structures so that the normal mucosal pattern can be easily identified. As shown in Fig. 1a, the vascular architecture in the gastric body appears hierarchically organized and honeycombed. The capillaries are coil-shaped and regular and of normal caliber. In the regular gastric mucosa, the fundus glands show round pits with round openings and the pyloric glands show continuous short rod-shaped pits with slit-like openings (Fig. 1a). The vascular pattern in non atrophic gastritis (g) resembles that of normal gastric mucosa, except for the presence of increased vessel tortuosity in the antral region and the occasional presence of leakage (Fig. 1b). In two patients, no vascular alterations were noted at all.

**Figure 2:**
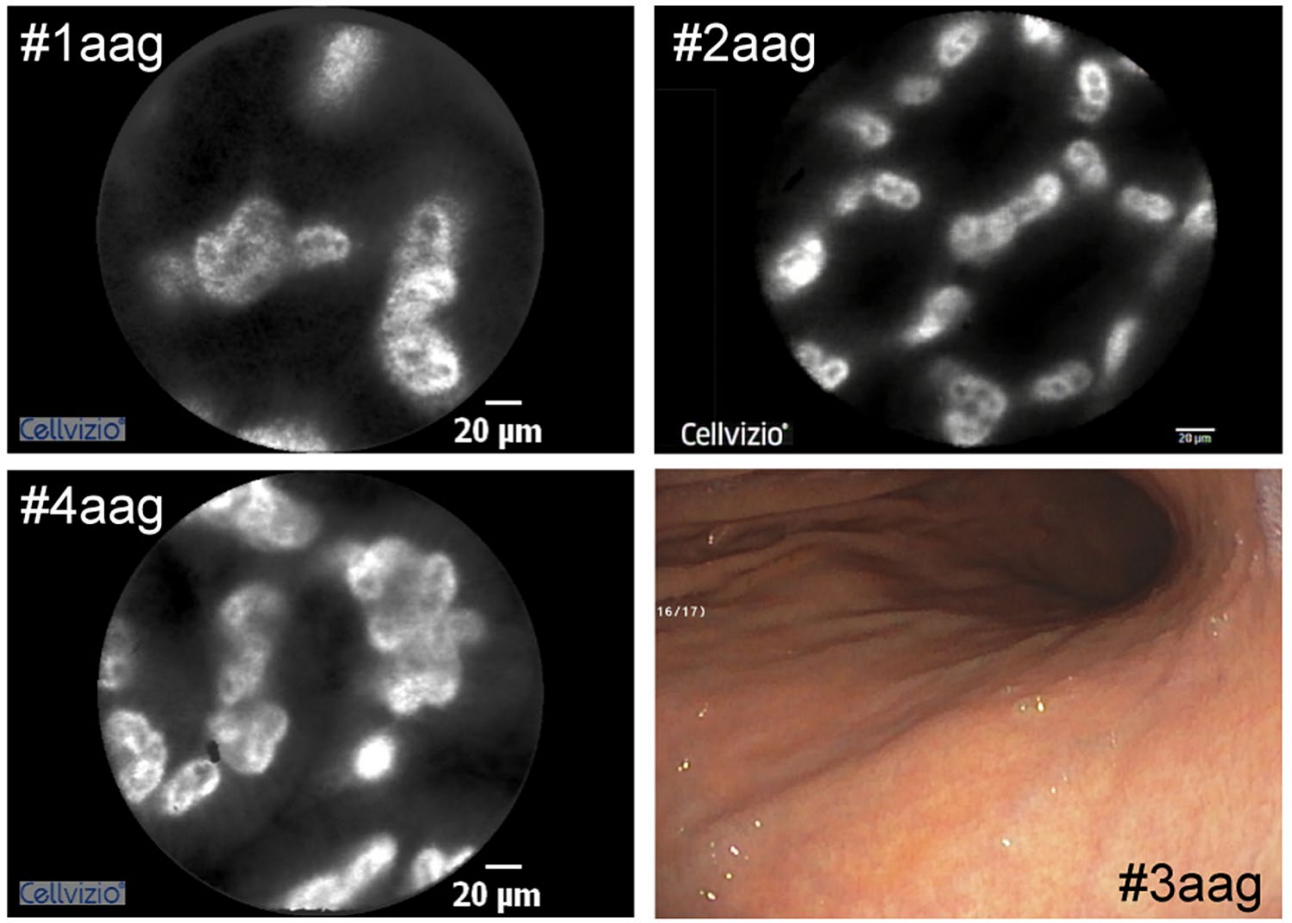
Representative pCLE images of the gastric mucosa of three patients (#1, #2 and #4) affected by atrophic autoimmune gastritis (aag). The endoscopy image of patient #3aag is shown as a representative example of standard atrophic mucosa.

Increased tortuosity and density of the vessels was the most representative feature of the mucosal vasculature in patients with atrophic autoimmune gastritis (Fig. 2). The coil-shaped capillaries surrounding the gastric pits were particularly tortuous and showed increased density in the antral region. The presence of a high number of leaky capillaries is a common, easily detectable trait in the inflamed areas^18^; however, we did not observe increased leakage in atrophic autoimmune gastritis, although occasional and mild vascular leakage was noted in one patient (#7aag). No other changes were observed.

**Figure 3:**
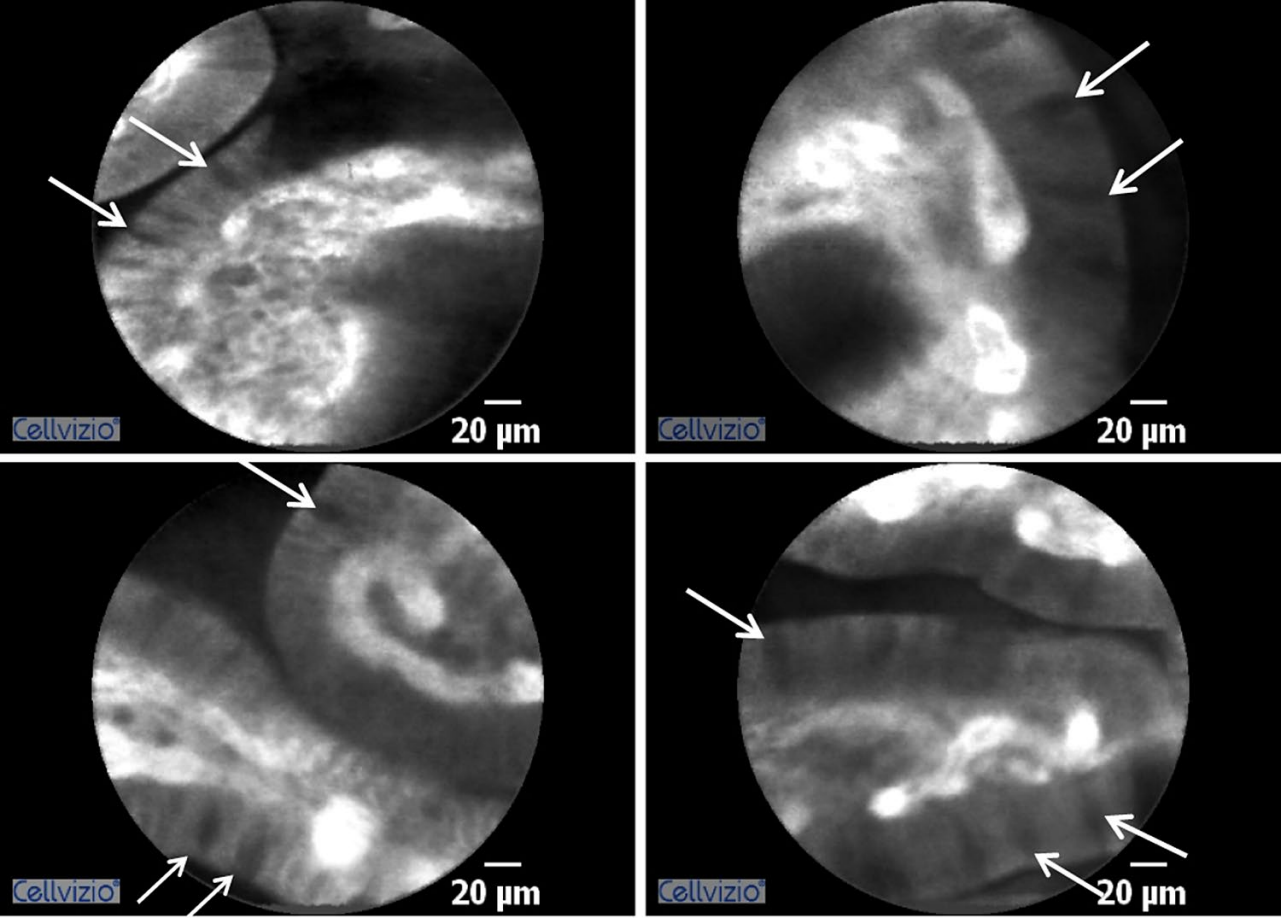
Representative pCLE images of the gastric mucosa of patients affected by atrophic metaplastic autoimmune gastritis (amg). White arrows indicate the presence of globet cells.

**Figure 4:**
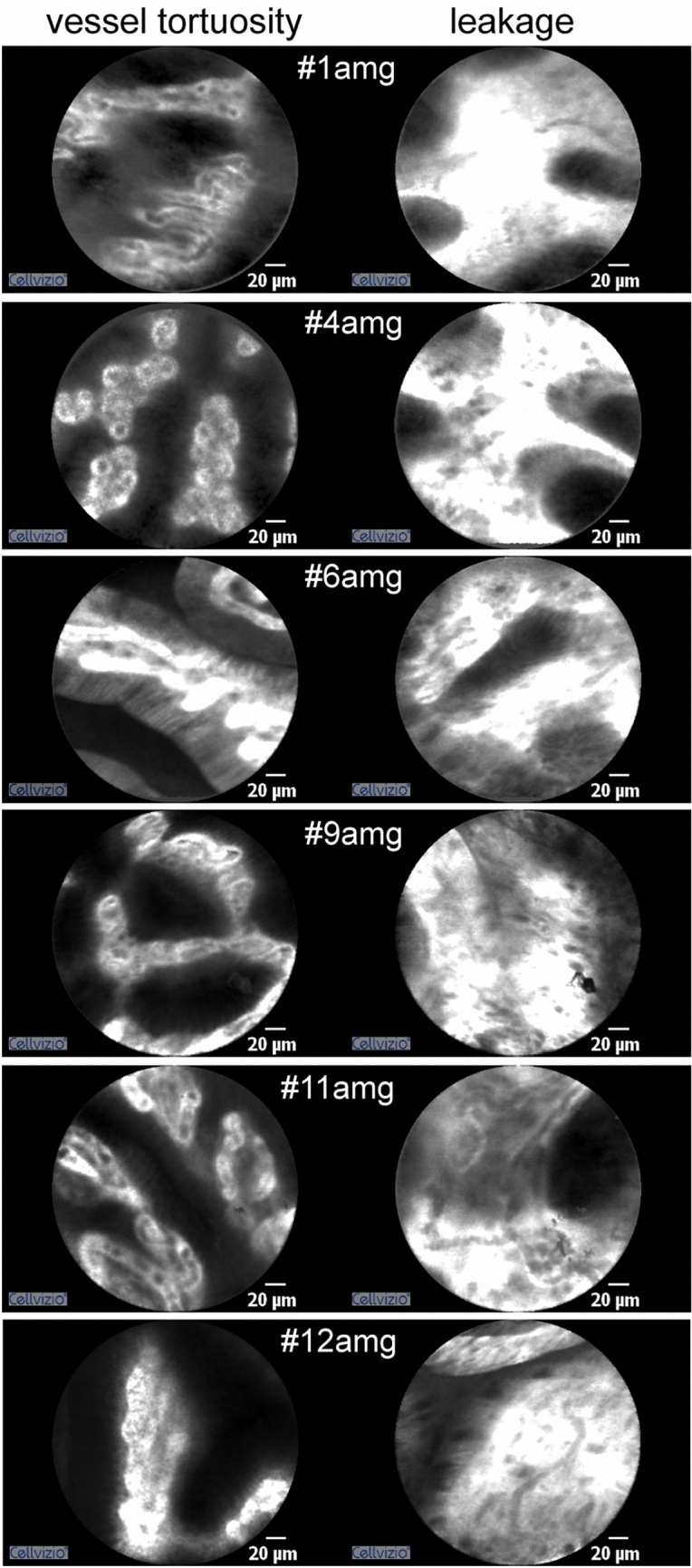
Vascular alterations detected by pCLE in patients affected by atrophic metaplastic autoimmune gastritis (amg). One image for each patient is representative of the presence of the typical tortuous coil-shaped vessels and the other of capillaries with elevated leakage.

In the group of patients with atrophic metaplastic autoimmune gastritis, the use of pCLE was also informative for the diagnosis, as the presence of goblet cells was easily detectable in all 12 cases studied (Fig. 3). Interestingly, pCLE detected an increased density of tortuous vessels with different spatial distribution and the presence of leakage, especially at the fundus and corpus, in all patients (Fig. 4).

**Figure 5:**
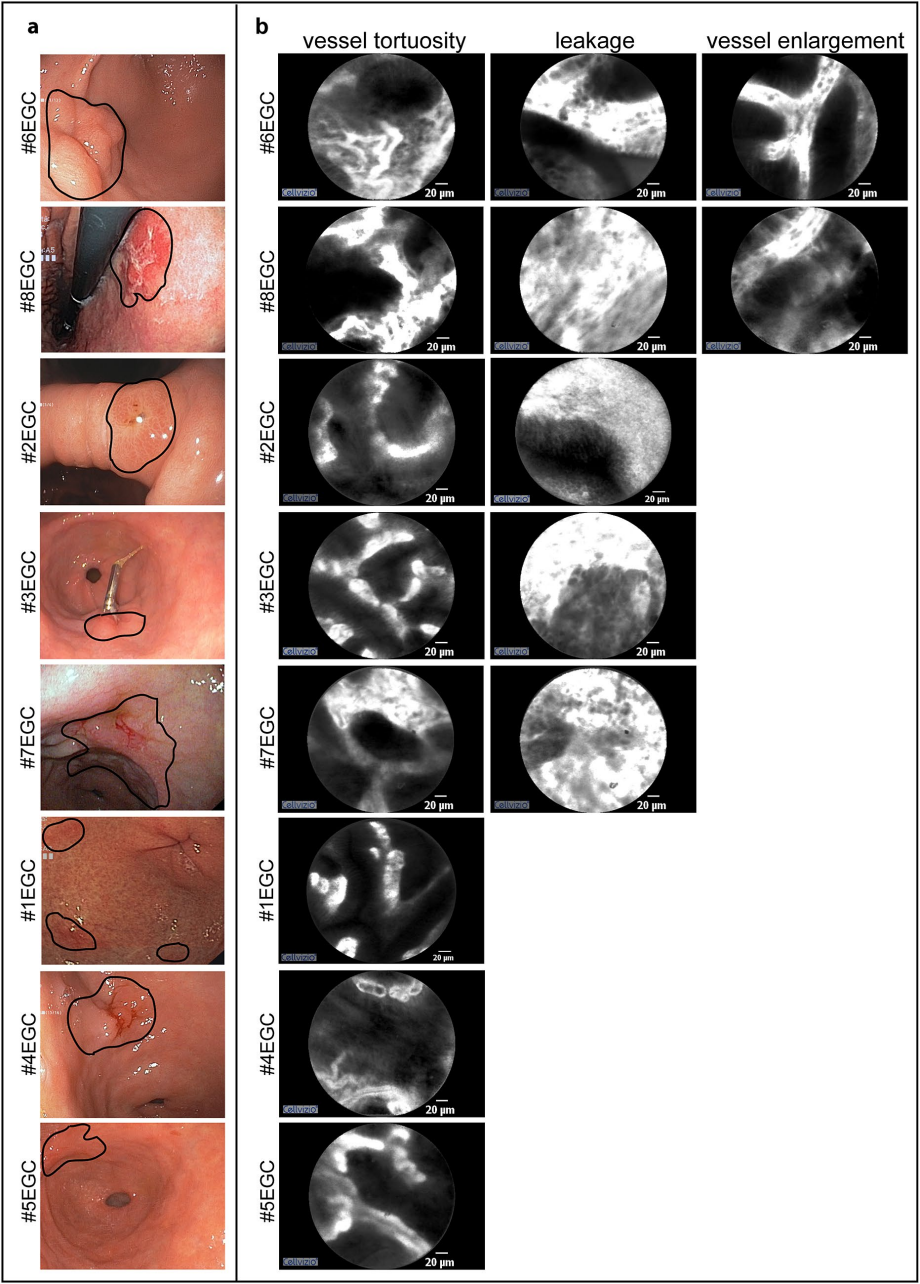
Endoscopic (**a**) and pCLE evaluation (**b**) in early gastric cancer (EGC) patients. The areas chosen for a diagnostic workup in endoscopic images are highlighted by a black line. The representative images of the vascular alterations for each patient are reported in **b**.

As highlighted in Fig. 5a (left panel, black line), endoscopy revealed the presence of dysplastic lesions or EGC in eight patients. All of these patients were further evaluated by pCLE to look for other possible dysplastic/neoplastic foci before endoscopic or surgical treatment was planned. The pCLE images of the mucosa of EGC patients were consistent with dysplastic or neoplastic conditions and single or multifocal neoplastic foci were detectable (Table 3). In one case, targeted pCLE-guided biopsy sampling was key to the diagnosis of EGC.

**Figure 6:**
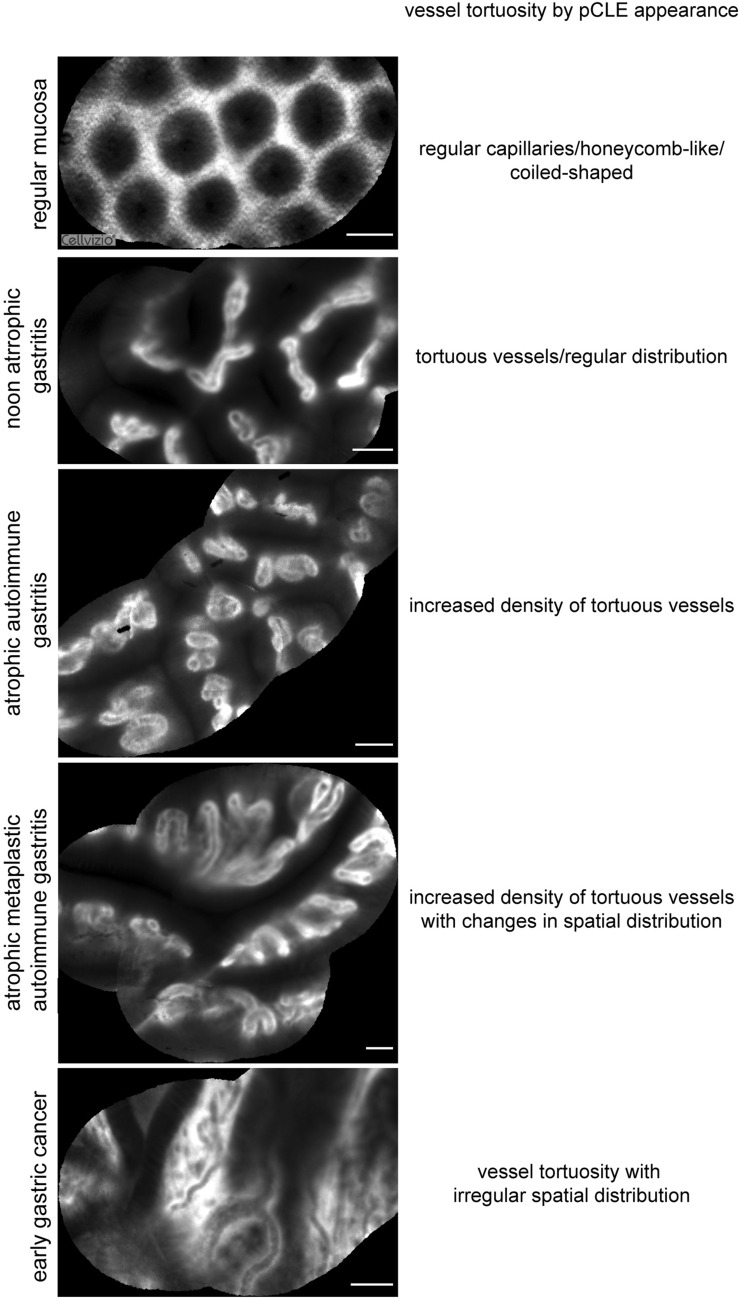
pCLE images of tortuous vessels. Representative mosaic reconstructions obtained from scanned panoramas of the gastric mucosa in healthy (regular mucosa), non atrophic gastritis, atrophic autoimmune gastritis, atrophic metaplastic autoimmune gastritis, and early gastric cancer (EGC) patients. The vessel tortuosity architecture documented by pCLE is described on the right for each patient category. The distribution of tortuous vessels is quite regular in atrophic autoimmune gastritis but in metaplastic patients such distribution is slightly changed. More unstructured vessels with an irregular spatial distribution can be appreciated in EGC patients. Scale bar = 50 μm.

According to the pCLE analyses the vascular changes in the surrounding normal mucosa were variable in EGC patients; however, vessel tortuosity was always present as a distinctive feature (Fig. 5b). Interestingly, tortuosity was characterized by an irregular spatial distribution of vessels (Fig. 6; for a comparison with “aag” and “amg” patients, see also Figs. 2, 4). Leakage was detectable in five patients and vessel enlargement in only two. Thus, the vascular pattern was not uniform, except for the presence of spatially disorganized and tortuous vessels, which was the common trait of EGC-associated vasculature.

A summary of the vascular alterations and mucosal architecture of all patients analyzed by pCLE can be found in Table 3.

## Discussion

A large body of research suggests that the use of pCLE may improve the detection of gastric lesions, including precancerous and intraepithelial neoplasia/EGC lesions, primarily due to its ability to assess tissue histology in vivo and enable real-time optical biopsy sampling^25–29^. pCLE was used by Li et al.^18^ to also analyze the vascular pattern in EGC and in precancerous lesions. They found that the introduction of a new classification based on the gastric pit patterns and the architecture of the vessels helped to distinguish malignant from benign lesions. In this classification, they described the presence of an increased number of capillaries with increased leakage in inflammatory gastric mucosa and the presence of irregular capillaries with heterogeneous leakage and expanded diameter in neoplasms^18^. More recently, pCLE was used to measure vessel diameter in a study aimed at defining quantitative diagnostic parameters for gastric atrophy^30^. Capillary diameter increased significantly in chronic gastritis with or without atrophy, but since the difference from normal gastric mucosa was too small, the authors felt that it was difficult to distinguish this change under real-time procedures. Recently, we thoroughly investigated the vascular features of locally advanced GC by evaluating the use of pCLE for the analysis of intratumoral angiogenesis, and we demonstrated that the functional and structural angiogenic parameters of the tumor blood network were fully detectable with this innovative endoscopic technique^20^. In this study, we provide evidence for the potential of this technique to assess vascular characteristics in gastric disease with malignant complications. Particular patterns of vascular changes are described that identify specific categories of patients with ECG or with disease at high risk for developing GC. The use of pCLE provided evidence that locally advanced GC is characterized by a remarkably abnormal and nonfunctional vasculature, regardless of tumor stage, location, and histological type^20^, suggesting that the vascular network may be a critical factor in establishing a compliant tumor microenvironment for this type of cancer, even in the early stages of tumor development. The formation of aberrant vasculature could favor or even precede lesions with malignant complications or higher GC risk and, together with other molecular and functional alterations of the microenvironment, profoundly influence cancer cell proliferation and migration as well as tumor progression^31,32^. Considering this possibility, we looked for the presence of vascular changes in areas that appeared regular on endoscopic view, even in patients at high risk for developing GC. There are several approaches to identify these patients, including noninvasive methods. Pre-endoscopic risk assessment is based on demographic and clinical characteristics, such as ethnicity, age, gender, smoking, and H. pylori status; in addition, further insight can be gained by serum concentrations of pepsinogen I and II and gastrin-17^6,33–36^. The analyses proposed in the present study may provide additional criteria for identifying and monitoring individuals at high risk for developing GC.

Our pCLE analyses suggest that gastritis was usually associated with increased vascularization characterized by a high number of tortuous vessels, and this feature was also the distinctive aspect of atrophic autoimmune disease. Nevertheless, the vessels were distributed in a spatially regular network. The same pattern of vessel tortuosity was observed in patients with atrophic metaplastic gastritis. However, the vascular density was higher and the spatial distribution of tortuous vessels was slightly irregular. Moreover, the presence of capillaries with increased leakage represented an additional and characteristic feature of atrophic metaplastic autoimmune gastritis. In these patients, the consistently increased vascular leakage could possibly be due to the presence of inflammatory cells and the release of factors that induce vascular permeability, such as VEGF-A.

Vascular features have already been considered in the diagnosis of EGC. The "vessel plus surface" classification system was used in imaging magnification endoscopy^10^ with the result that EGC included the presence of a clear demarcation line between cancerous and noncancerous mucosa and the presence of an irregular microvascular pattern within the demarcation line, recognizable by the abrupt change in this pattern between lesion and nonlesion areas^10^. In our study, we were able to detect vascular changes even in non-lesion areas using pCLE, supporting the evidence that vascular changes may indicate a favorable microenvironment for carcinogenesis. We found that the mucosal vasculature of EGC patients was also characterized by vessel tortuosity, but in contrast to the regular organization observed in atrophic gastritis, these vessels often appeared patchy, as is also the case in neoplastic gastric tissue^20^. This peculiar aspect of vessel tortuosity was also found, in part, in atrophic metaplastic autoimmune gastritis. Thus, our results suggest that the presence of tortuous vessels with a slightly irregular spatial distribution associated with vascular leakage in precancerous conditions may be of prognostic value in identifying patients at high risk for developing GC.

The vascular architecture described in this study represents a novel and accurate criterion for classifying precancerous conditions at high risk for developing GC. In particular, the density of tortuous vessels may distinguish different categories within gastritis, while their irregular distribution is a distinctive feature of EGC. Thus, the use of pCLE technology in the classification of gastric diseases with a detailed description of vascular features may provide further insights than the mere differentiation between malignant and benign lesions, as reported by Li et al.^18^.

Recently, the use of advanced endoscopic techniques for proper gastric biopsy sampling has been shown to shorten the diagnostic delay, especially in early autoimmune gastritis^4^. Therefore, the use of pCLE in general could be a useful technical support in a surveillance program. Most importantly, the standard protocol Sydney-Houston, based on antrum biopsies, is not suitable for the detection of atrophic autoimmune gastritis confined to the corpus^37^. In this case, the vascular alterations detected by pCLE might actually represent a valid parameter for performing screening of patients.

To our knowledge, this is the first report demonstrating that the use of pCLE can be a valid endoscopic strategy to better determine risk. Unfortunately, the ability to perform only qualitative analyses in a small number of patients is a major limitation. However, we believe that this study may suggest new avenues for the use of an endoscopic technique that we believe can provide not only structural but also functional evidence of the diseased gastric tissue, thus expanding the possibilities for improving diagnosis and prognosis. Although there may be considerable variability in the interpretation of images with respect to the extent of vascular alterations, we are confident that pCLE is a viable technique to distinguish the abnormalities of the vasculature associated with tumors or pre-tumoral lesions characterized by normal mucosa from the hierarchical organization of normal vessels. Taken together, these results suggest that the use of pCLE may open new perspectives for a better understanding of the role of vascular properties in influencing the biological behavior of gastric diseases.

## Methods

### Patients

For this study, we consecutively enrolled 42 patients who underwent endoscopy at the Oncological Gastroenterology Division of the CRO-IRCCS, National Cancer Institute of Aviano (PN), Italy for diagnostic working up from January 2019 to December 2020. According to the histology they were classified as healthy control cases with normal (n) mucosa, autoimmune gastritis without atrophy (g), atrophic autoimmune gastritis (aag), atrophic metaplastic autoimmune gastritis (amg), early gastric cancer (EGC). Tables 1 and 2 summarize patients’ classification. All the patients signed the informed consent form to undergo the pCLE endomicroscopic analyses. All procedures were in accordance with the ethical standards of the responsible committee on human experimentation (institutional and national) and with the Helsinki Declaration of 1964 and later versions. The study was approved by the Institutional Board of the CRO-IRCCS (IRB no. CRO-2014-03), Aviano, Italy.

### Endoscopy procedures and pCLE analyses

Gastric mucosa was examined with high definition (HD) white light and narrow-band imaging (NBI) to improve visibility of blood vessels and mucosal structures. pCLE analyses were performed with a GastroFlex UHD probe (Cellvizio, Mauna Kea Technology, Paris, France) during upper gastrointestinal endoscopy (Olympus H180 and H190 series). pCLE was applied to areas of gastric mucosa with a raised, depressed, or discolored appearance, as well as to the regular mucosa of the antrum, angulus, antrum/corpus border, lesser and greater curvature of the body, and cardia, followed by conventional biopsy specimens taken at the end of the examination. EGC and dysplastic lesions were classified according to the Paris classification of early and/or superficial tumors in the gastrointestinal tract^24^. Images were recorded within the first 10 min after i.v. injection of fluorescein (3 ml of a 10% solution). pCLE images were acquired at 12 frames per second to ensure high video quality and direct visualization at the level of individual erythrocytes. pCLE acquisitions were performed for at least 3 min, resulting in real-time imaging of more than 2000 images. Reconstructions of the scanned panoramic mucosal images were created using the video mosaic function of the analysis software. The mucosal architecture, presence of leakage, tortuosity and enlargement of vessels, and efficiency of blood flow were assessed as previously described^21^. In particular, to better characterize the presence of vessel tortuosity, in addition to applying a criterion that is undoubtedly morphological due to its conceptual simplicity, we used the ratio between the meandering vessel length and the rectilinear distance between its endpoints. At least 50 vessels were analyzed. Vessel tortuosity was established as a characteristic of vasculature when an average value of 1.5 was found. Vessel enlargement was defined for those dilated capillaries whose diameter was greater than 15 µm. Images were stored digitally and reviewed using the dedicated software package (Cellvizio Viewer, Mauna Kea Technologies) by a highly experienced investigator (PS) who was blinded to any clinical, endoscopic, or histopathologic information, and independently postanalyzed by an endoscopist (MF).

### Histology

For histological examination, biopsy specimens were fixed in buffered formalin 10%, embedded in paraffin and stained with hematoxylin and eosin using the modified Giemsa method for *H. pylori*. OLGA-OLGIM staging was used to define gastritis^23^, assessing the following structural variables: activity (amount of neutrophil infiltration), inflammation (amount of mononuclear-cell infiltration), atrophy (loss of glandular tissue), intestinal metaplasia, and *H. pylori* density. The diagnosis of autoimmune gastritis was based on anti-parietal cell antibody (APCA) positivity. GC was classified according to Lauren classification^38^ and the disease stage was assessed according to TNM criteria.
